# Supplementary material for: Bayesian hidden mark interaction model for detecting spatially variable genes in imaging-based spatially resolved transcriptomics data
Source: Front Genet. 2024 Apr 25;15:1356709. doi: 10.3389/fgene.2024.1356709 (PMC11079231; doi:10.3389/fgene.2024.1356709)
Supplement: Supplementary file 1 [file DataSheet1.PDF]

Supplementary Material

1 MCMC ALGORITHM APPENDIX

The model parameter space consists of $(\boldsymbol{H}, \boldsymbol{M}, \boldsymbol{\Phi}, \Xi, \boldsymbol{\omega}_0, \boldsymbol{\theta})$, where $\boldsymbol{H} = \{\eta_{ij}, i = 1, \cdots, n, j = 1, \cdots, p\}$ is the extra zero indicator matrix, $\boldsymbol{M} = \{\mu_{0j}, \mu_{1j}, j = 1, \cdots, p\}$ is the collection of group mean parameters for all genes, $\boldsymbol{\Phi} = \{\phi_{0j}, \phi_{1j}, j = 1, \cdots, p\}$ is the collection of dispersion parameters for all genes, $\Xi = \{\xi_{ij}, i = 1, \cdots, n, j = 1, \cdots, p\}$ is the collection of gene expression level indicator, $\boldsymbol{\omega}_0 = \{\omega_{0j}, j = 1, \cdots, p\}$ is the first-order intensity parameter in the modified energy function, $\boldsymbol{\theta} = \{\theta_j, j = 1, \cdots, p\}$ is the interaction parameter in the modified energy function. The full posterior is,

$$p(\boldsymbol{H}, \boldsymbol{M}, \boldsymbol{\Phi}, \Xi, \boldsymbol{\omega}_0, \boldsymbol{\theta}|Y) \propto f(Y|\boldsymbol{H}, \Xi, \boldsymbol{M}, \boldsymbol{\Phi})p(\boldsymbol{H})p(\boldsymbol{M}|\Xi)p(\boldsymbol{\Phi}|\Xi)p(\Xi|\boldsymbol{\omega}_0, \boldsymbol{\theta})\pi(\boldsymbol{\omega}_0)\pi(\boldsymbol{\theta}).$$

As stated in Section 3 in the main text, the full likelihood and priors are:

$$f(\boldsymbol{Y}|\boldsymbol{H}, \Xi, \boldsymbol{M}, \boldsymbol{\Phi}) = \prod_{i=1}^{n} \prod_{j:\xi_{ij}=0,\eta_{ij}=0} \text{NB}(y_{ij}; s_i\mu_{0j}, \phi_{0j}) \prod_{j:\xi_{ij}=1,\eta_{ij}=0} \text{NB}(y_{ij}; s_i\mu_{1j}, \phi_{1j}),$$

$$\pi(\boldsymbol{H}) = \prod_{i=1}^{n}\prod_{j=1}^{p} \text{Be-Bern}(\eta_{ij}; a_\pi, b_\pi),$$

$$\pi(\boldsymbol{M}|\Xi) = \prod_{i=1}^{n} \prod_{j:\xi_{ij}=0} \text{Ga}(\mu_{0j}; a_\mu, b_\mu) \prod_{j:\xi_{ij}=1} \text{Ga}(\mu_{1j}; a_\mu, b_\mu),$$

$$\pi(\boldsymbol{\Phi}|\Xi) = \prod_{i=1}^{n} \prod_{j:\xi_{ij}=0} \text{Ga}(\phi_{0j}; a_\phi, b_\phi) \prod_{j:\xi_{ij}=1} \text{Ga}(\phi_{1j}; a_\phi, b_\phi),$$

and as described in Section 2.3, the priors in the hidden Bayesian mark interaction model are:

$$\pi(\Xi|\boldsymbol{\omega}_0, \boldsymbol{\theta}) = \prod_{j=1}^{p} \pi(\boldsymbol{\xi}_{\cdot j}|\omega_{0j}, \theta_j),$$

$$\pi(\boldsymbol{\omega}_0) = \prod_{j=1}^{p} \text{N}(\omega_{0j}; \mu_\omega, \tau_\omega^2),$$

$$\pi(\boldsymbol{\theta}) = \prod_{j=1}^{p} \text{N}(\theta_j; \mu_\theta, \tau_\theta^2),$$

where the full formulation of $\pi(\boldsymbol{\xi}_{\cdot j}|\omega_{0j}, \theta_j)$ is shown in Equation (4) in the main text. The p.d.f's of the involved common distributions are given below:

$$\text{If } x \sim \text{NB}(\mu, \phi), \text{ then } p(x) = \frac{\Gamma(x+\phi)}{x!\Gamma(\phi)} \left(\frac{\phi}{\mu+\phi}\right)^{\phi} \left(\frac{\mu}{\mu+\phi}\right)^{x},$$

$$\text{If } x \sim \mathrm{N}(\mu, \sigma^2), \text{ then } p(x) = \frac{1}{\sqrt{2\pi}\sigma} \exp\left(-\frac{(x-\mu)^2}{2\sigma^2}\right),$$

$$\text{If } x \sim \mathrm{Ga}(\alpha, \beta), \text{ then } p(x) = \frac{\beta^\alpha}{\Gamma(\alpha)} x^{\alpha-1} \exp(-\beta x),$$

$$\text{If } x \sim \text{Be-Bern}(a, b), \text{ then } p(x) = \frac{1}{a+b} \frac{\Gamma(a+x)\Gamma(b+1-x)}{\Gamma(a)\Gamma(b)}.$$

Our research interest is to identify SVGs through estimating $\boldsymbol{\theta}$. To estimate $\boldsymbol{\theta}$, all parameters $(\boldsymbol{H}, \boldsymbol{M}, \boldsymbol{\Phi}, \Xi, \boldsymbol{\omega}_0, \boldsymbol{\theta})$ are proposed and estimated in the MCMC algorithm. We use a random walk Metropolis-Hastings (RWMH) algorithm to estimate $\boldsymbol{M}$ and $\boldsymbol{\Phi}$. Due to the intractable normalizing constant in Equation (4), parameters in the hidden Bayesian mark interaction model are estimated by Double Metropolis-Hastings (DMH) algorithm. And $\boldsymbol{H}$ and Ξ are estimated via Gibbs sampler. MCMC algorithm is implemented gene-wisely. The following metropolis-hastings algorithm is described for each gene $j, j = 1, \cdots, p$.

1.1 Random walk Metropolis-Hastings algorithm

Update of group mean $\boldsymbol{M}$: We update μ_{0j} and μ_{1j} separately, but with the same procedure. Thus, the updating mechanism of μ_{0j} and μ_{1j} are integrated as updating μ_{kj}. We propose a new μ^*_{kj} from $\mathrm{Ga}(a_\mu, b_\mu)$, the proposed μ^*_{kj} will be accepted with probability $\min(1, r)$. The Hastings ratio r is

$$r = \prod_{i:\eta_{ij}=0,\xi_{ij}=k} \frac{\mathrm{NB}(y_{ij}; s_i\mu^*_{kj}, \phi_{kj})\mathrm{Ga}(\mu^*_{kj}; a_\mu, b_\mu)J(\mu_{kj}; \mu^*_{kj})}{\mathrm{NB}(y_{ij}; s_i\mu_{kj}, \phi_{kj})\mathrm{Ga}(\mu_{kj}; a_\mu, b_\mu)J(\mu^*_{kj}; \mu_{kj})}.$$

Note that the proposal density ratio cancels out for this RWMH update.

Update of dispersion parameter $\boldsymbol{\Phi}$: Similar to updating $\boldsymbol{\mu}$, we update ϕ_{0j} and ϕ_{1j} separately, but with the same procedure. Similarly, the updating mechanism of ϕ_{0j} and ϕ_{1j} are summarized as updating ϕ_{kj}. We propose a new ϕ^*_{kj} from $\mathrm{Ga}(a_\phi, b_\phi)$, the proposed ϕ^*_{kj} will be accepted with probability $\min(1, r)$. The Hastings ratio r is

$$r = \prod_{i:\eta_{ij}=0,\xi_{ij}=k} \frac{\mathrm{NB}(y_{ij}; s_i\mu_{kj}, \phi^*_{kj})\mathrm{Ga}(\phi^*_{kj}; a_\phi, b_\phi)J(\phi_{kj}; \phi^*_{kj})}{\mathrm{NB}(y_{ij}; s_i\mu_{kj}, \phi_{kj})\mathrm{Ga}(\phi_{kj}; a_\phi, b_\phi)J(\phi^*_{kj}; \phi_{kj})}.$$

Note that the proposal density ratio cancels out for this RWMH update.

1.2 Double Metropolis-Hastings algorithm

Update of first-order intensity parameter ω_{0j}: We first propose a new ω^*_{0j} from $\mathrm{N}(\omega_{0j}, \tau^2_\omega)$. We implement the Gibbs sampler to simulate an auxiliary variable $\boldsymbol{\xi}^*_{\cdot j}$ starting from $\boldsymbol{\xi}_{\cdot j}$ based on the new ω^*_0. The proposed value ω^*_{0j} is accepted to replace the old value with probability $\min(1, r)$. The Hastings ratio r is given as

$$r = \frac{\pi(\boldsymbol{\xi}^*_{\cdot j}|\omega_{0j}, \omega_{1j}, \theta_j)\pi(\boldsymbol{\xi}_{\cdot j}|\omega^*_{0j}, \omega_{1j}, \theta_j)\mathrm{N}(\omega^*_{0j}; \mu_\omega, \tau^2_\omega)J(\omega_{0j}; \omega^*_{0j})}{\pi(\boldsymbol{\xi}_{\cdot j}|\omega_{0j}, \omega_{1j}, \theta_j)\pi(\boldsymbol{\xi}^*_{\cdot j}|\omega^*_{0j}, \omega_{1j}, \theta_j)\mathrm{N}(\omega_{0j}; \mu_\omega, \tau^2_\omega)J(\omega^*_{0j}; \omega_{0j})}.$$

Update of the interaction parameter θ_j: We first propose a new θ_j^* from $\mathrm{N}(\theta_j, \tau_\theta^2)$. We implement the Gibbs sampler to simulate an auxiliary variable $\boldsymbol{\xi}_j^*$ starting from $\boldsymbol{\xi}_j$ based on the new θ_j^*. The proposed value θ_j^* is accepted to replace the old value with probability $\min(1, r)$. The Hastings ratio r is

$$r = \frac{\pi(\boldsymbol{\xi}_{\cdot j}^*|\omega_{0j}, \omega_{1j}, \theta_j)\pi(\boldsymbol{\xi}_{\cdot j}|\omega_{0j}, \omega_{1j}, \theta_j^*)\mathrm{N}(\theta_j^*; \mu_\theta, \tau_\theta^2)J(\theta_j; \theta_j^*)}{\pi(\boldsymbol{\xi}_{\cdot j}|\omega_{0j}, \omega_{1j}, \theta_j)\pi(\boldsymbol{\xi}_{\cdot j}^*|\omega_{0j}, \omega_{1j}, \theta_j^*)\mathrm{N}(\theta_j; \mu_\theta, \sigma_\theta^2)J(\theta_j^*; \theta_j)}.$$

1.3 Gibbs sampler

Update of zero-inflation indicator $\boldsymbol{H}$: The Gibbs sampler is implemented to estimate each $\eta_{ij}, i = 1, \cdots, n$ that corresponds to $y_{ij} = 0$,

$$p(\eta_{ij}|\cdot) \propto \left(\mathrm{NB}(y_{ij}; s_i\mu_{1j}, \phi_{1j})^{1-\eta_{ij}}\right)^{\xi_{ij}} \left(\mathrm{NB}(y_{ij}; s_i\mu_{0j}, \phi_{0j})^{1-\eta_{ij}}\right)^{1-\xi_{ij}} \times \text{Be-Bern}(\eta_{ij}; a_\pi, b_\pi),$$

$$\eta_{ij}|\xi_{ij} = k, \cdot \sim \text{Bern}\left(\frac{\pi(\eta_{ij} = 1|\xi_{ij} = k, \cdot)}{\pi(\eta_{ij} = 1|\xi_{ij} = k, \cdot) + \pi(\eta_{ij} = 0|\xi_{ij} = k, \cdot)}\right).$$

Update of gene expression level indicator $\boldsymbol{\xi}$: New ξ_{ij} is generated from $\mathrm{Bern}(p_i)$, where $p_i = \frac{\pi(\xi_{ij}=1)}{\pi(\xi_{ij}=1)+\pi(\xi_{ij}=0)}$. In our implementation, we calculated p_i by $p_i = \frac{1}{\exp(\log(\pi(\xi_{ij}=0))-\log(\pi(\xi_{ij}=1)))+1}$, where $\pi(\xi_{ij} = 1)$ is given in Equation (7) in the main text and $\pi(\xi_{ij} = 0)$ is calculated in the same way.

2 SCALABILITY TEST

The scalability test was conducted in R with Rcpp package on a computer equipped with Intel Xeon Platinum 9242 Processor. Within each MCMC iteration, for each gene j, we need to update $\mu_{0j}, \mu_{1j}, \phi_{0j}, \phi_{1j}, \boldsymbol{\xi}_j, \omega_{0j}, \theta_j$, and $\boldsymbol{\eta}_j$. The total number of parameters is $6 + 2n$.

To empirically test the scalability, we generated simulated datasets with $p = 100$ and six different number of spots: $n = 100$, 225, 400, 625, and 900, following the data generation scheme proposed by Jiang et al. (2022) (also detailed in Section S4). For each scenario, we generated ten replicates, each featuring spot pattern and around 30% false zeros. We ran an MCMC chain with 1,000 iterations for each dataset.

Figure S1 demonstrates the runtime of the MCMC algorithm as a function of the number of spots. It is obvious that the algortihm is fast when number of spot is low. For example, the runtime of $n = 100$ spots is as short as 26.42s on average. Further, the actual runtime increases approximately linear in n, which is consistent with the theoretical time complexity. To further evaluate the relationship between running time and number of spots, we fit a linear regression for the runtime (per 1,000) iterations versus n. The resulting regression model has an adjusted R^2 of 0.942, which indicates that the runtime depends mainly on number of spots n. The estimated regression model is running time $= -657.449 + 3.748n$.

3 SENSITIVITY ANALYSIS

A comprehensive sensitivity analysis was performed to assess the robustness of BOOST-HMI against variations in the hyperparameters τ_θ and τ_ω. For this analysis, we applied BOOST-HMI to ten replicates within a scenario characterized by a medium level of zero-inflation (30% false zeros). We systematically explored six values for τ_θ, spanning from 0.2 to 4, and for τ_ω, from 0.2 to 10, generating a total of 36 distinct hyperparameter combinations.

The results, shown in Figure S2, revealed that BOOST-HMI maintains consistent performance across a wide range of hyperparameter settings, as indicated by stable AUC values. Specifically, despite the extensive variation in τ_θ and τ_ω, the variations in AUC scores were minimal, demonstrating that BOOST-HMI's performance is largely invariant to the choice of these hyperparameters. This robustness suggests that BOOST-HMI can be reliably applied across different settings without the need for extensive hyperparameter tuning.

4 SIMULATION STUDY WITH MODEL MIS-SPECIFICATION

To examine the robustness and generalizability of BOOST-HMI, we tested our model on simulated data that were not from our model itself. Specifically, we followed Jiang et al. (2022) to generate simulated data from two artificial spatial patterns, termed "spot" and "linear" on a 16×16 square lattice, totaling $n = 256$ cells. We set $p = 100$ genes, of which 15 were true SVGs. For each gene j, the log relative expression level at cell i was generated *via*

$$\log \tilde{y}_{ij} = \begin{cases} \beta_0 + e_i + \epsilon_{ij} & \text{if gene } j \text{ is an SVG} \\ \beta_0 + \epsilon_{ij} & \text{if gene } j \text{ is a non-SVG} \end{cases},$$

where β_0 denotes the baseline relative expression level and ϵ_{ij} denotes the non-spatial errors following $\mathrm{N}(0, \sigma_\epsilon^2)$. We set $\beta_0 = 2$ and $\sigma_\epsilon = 0.3$. For a non-SVG, the relative expression levels were from a log-normal (LN) distribution with mean and variance being 2 and 0.3^2. Consequently, no spatial correlation should be observed. For an SVG with the spot pattern, the values of e_i's of the four center cells at $(8, 8)$, $(8, 9)$, $(9, 8)$, and $(9, 9)$ were set to $\log 6$, while all others were linearly decreased to zero within a radius of five cells. For an SVG with the linear pattern, the value of e_i of the most bottom-left cell at $(1, 1)$ was set to $\log 6$, while all others were linearly decreased to zero along the diagonal line. To mimic the excess zeros and over-dispersion, we sampled each gene expression count y_{ij} from a ZINB model, $y_{ij} \sim \pi_i I(y_{ij} = 0) + (1 - \pi_i)\mathrm{NB}(s_i \tilde{y}_{ij}, \phi_j)$, where the size factor $s_i \sim \mathrm{LN}(0, 0.2^2)$ and the dispersion parameter ϕ_j was from an exponential distribution with mean 10. For the choice of the false zero proportion π_i, we randomly selected 10%, 30%, or 50% counts and forced their values to zero. Combined with the two patterns and three zero-inflation settings, there were six different scenarios. For each scenario, we repeated the above steps to generate ten replicates.

Model performance was evaluated using the AUC metric. Figure S3 depicted the AUC achieved by BOOST-HMI and various competing methods under the six scenarios. In scenarios characterized by low sparsity, SPARK achieved the highest AUC values. However, BOOST-HMI and BOOST-GP exhibited similar levels of performance. As the prevalence of excess zeros increased, both BOOST-HMI and BOOST-GP showed improved effectiveness, with BOOST-GP taking the lead due to its model assumptions being more closely aligned with the data generation process. Notably, BOOST-HMI consistently surpassed both BinSpect and SpatialDE across all tested scenarios.

In summary, BOOST-HMI demonstrates strong performance even in the face of model mis-specification, underscoring its robustness and generalizability.

5 STATISTICAL POWER ANALYSIS

We conducted a simulation study to compare the model's statistical power and its ability to control false discoveries. We generated $p = 12,000$ genes, among which 10% are SVGs. Statistical power and Type I

error are illustrated in Figure S4. BOOST-HMI has the highest statistical power, and SPARK-X has the best ability to control false discoveries with a type I error of zero.

The implications of these results suggest that the choice between BOOST-HMI and SPARK-X may depend on the specific objectives. When identifying true SVGs is important, BOOST-HMI is a compelling option. If the priority is to avoid false discoveries, SPARK-X is a better model.

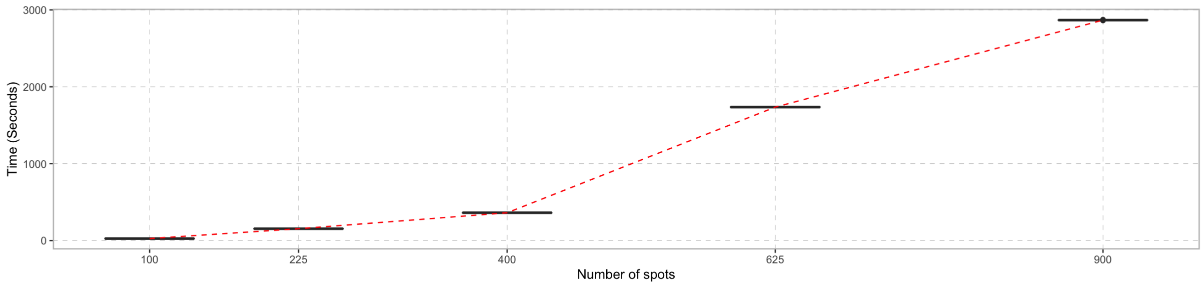

Figure S1: Scalability test: The boxplots of the runtime in seconds (per 1,000 MCMC iterations) in terms of the number of spots.

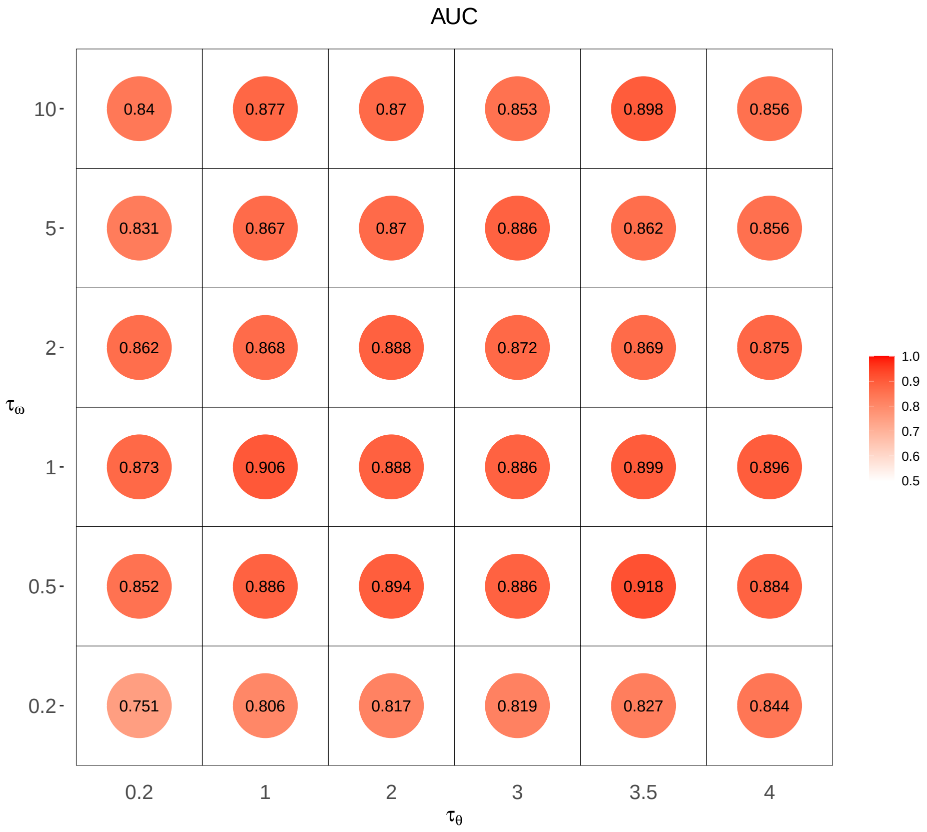

Figure S2: Sensitivity analysis: The heatmaps of averaged AUCs achieved by different hyperparameters τ_ω and τ_θ.

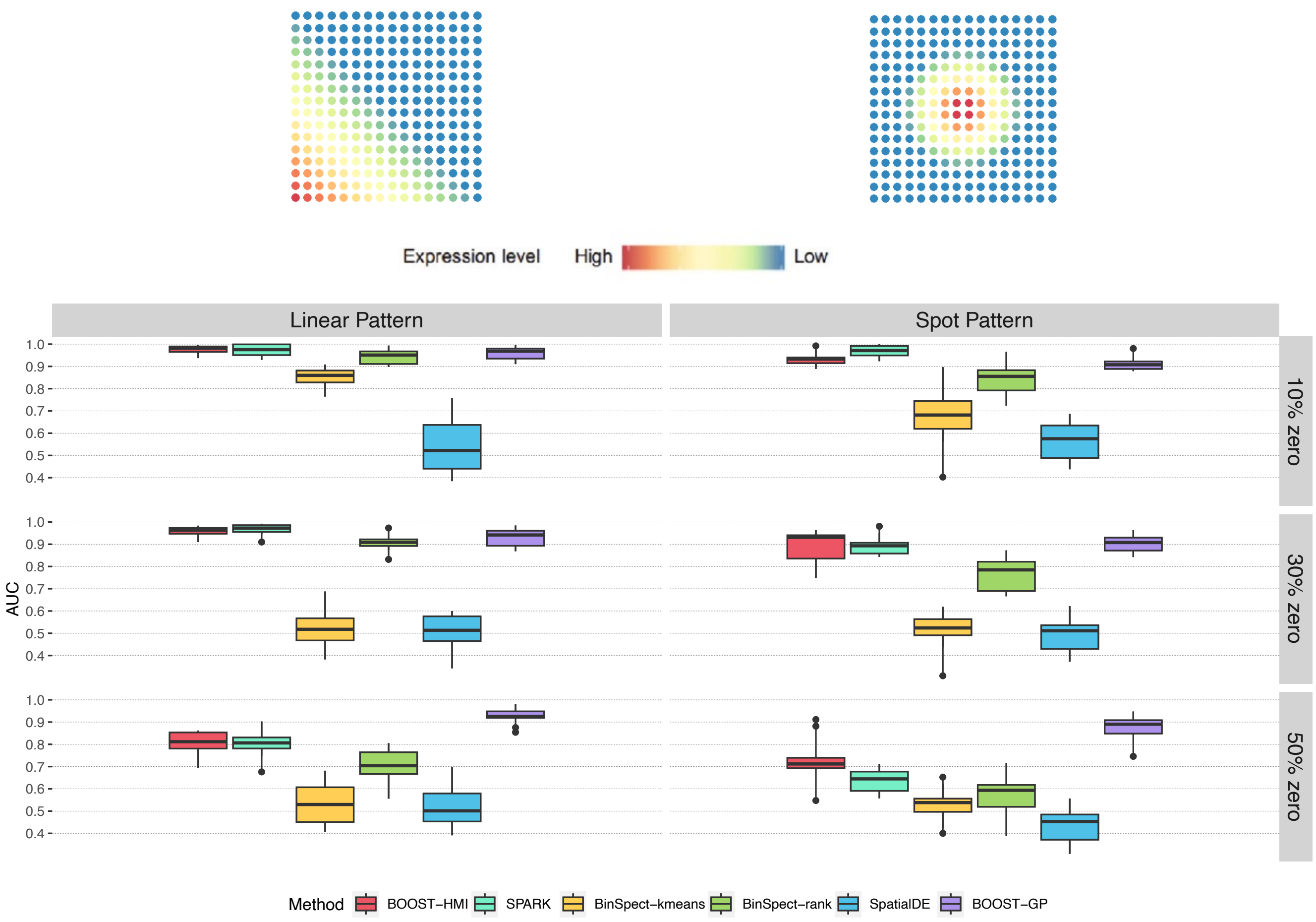

Figure S3: Simulation study with model mis-specification: The boxplots of AUCs achieved by BOOST-HMI, SPARK, SpatialDE, BinSpect-kmeans, BinSpect-rank and BOOST-GP across 6 scenarios.

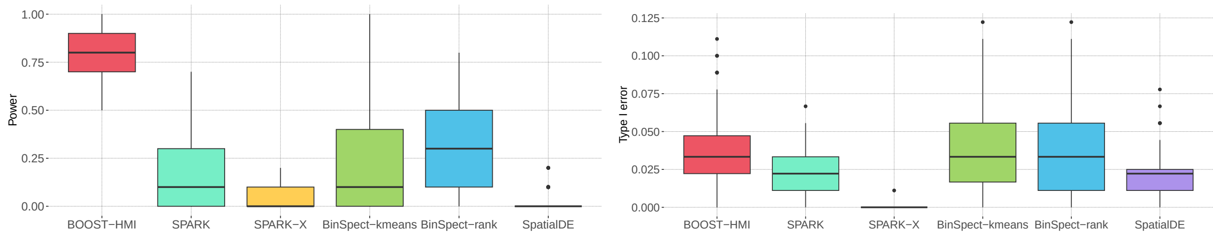

Figure S4: Statistical power analysis: The boxplots of statistical power and type I error rate achieved by BOOST-HMI, SPARK, SPARK-X, BinSpect-kmeans, BinSpect-rank, and SpatialDE.

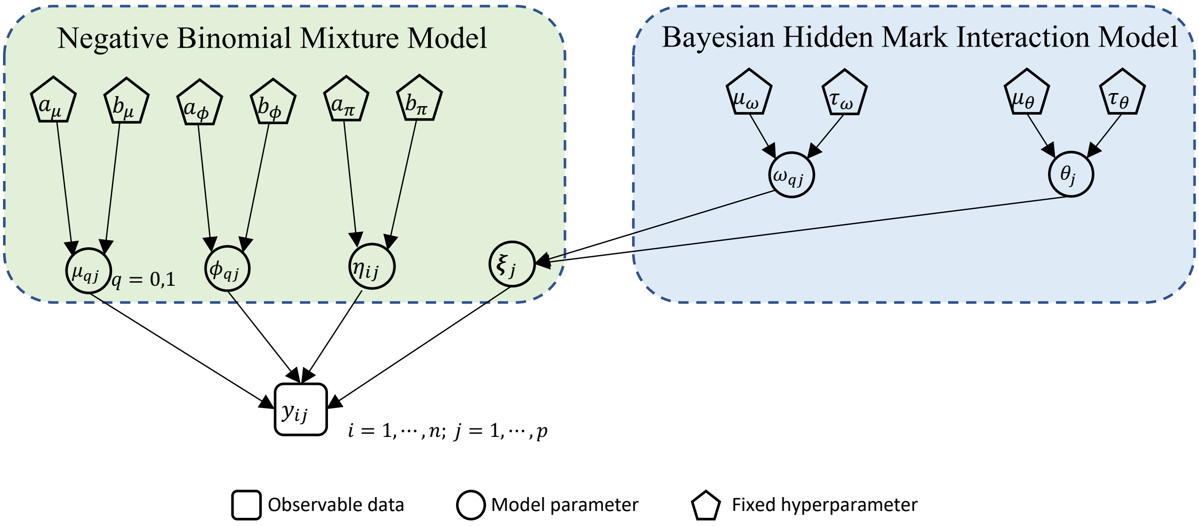

Figure S5: A graphical representation of BOOST-HMI for identifying SVGs. Nodes in square, circle, and hexagon refer to observable data, model parameter, and fixed hyperparameter, respectively. The link between two nodes represents a direct probabilistic dependence.

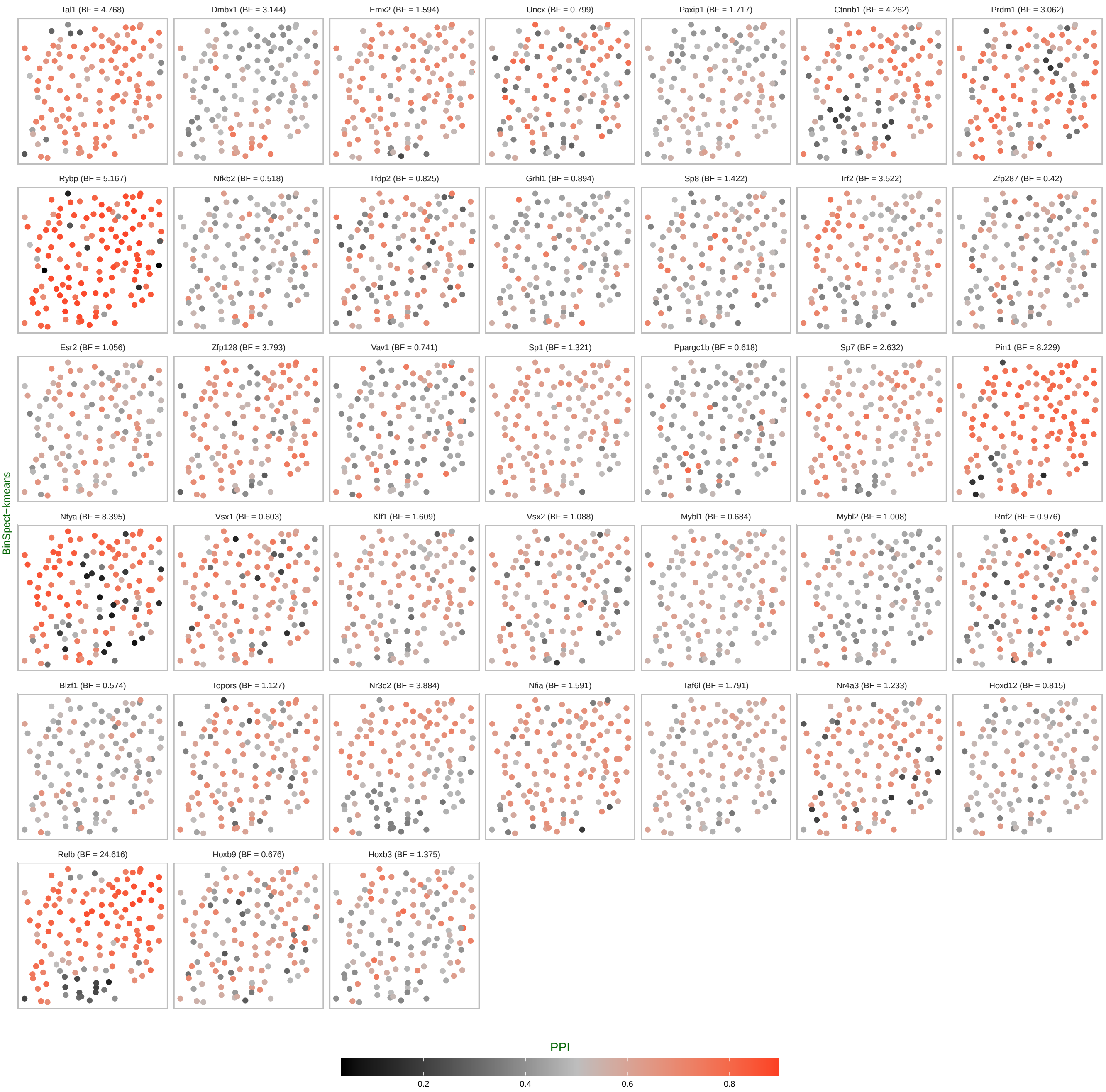

Figure S6: Mouse hippocampus seqFISH data: Spatial pattern of hidden gene expression indicator of SVGs detected by BinSpect-kmeans.

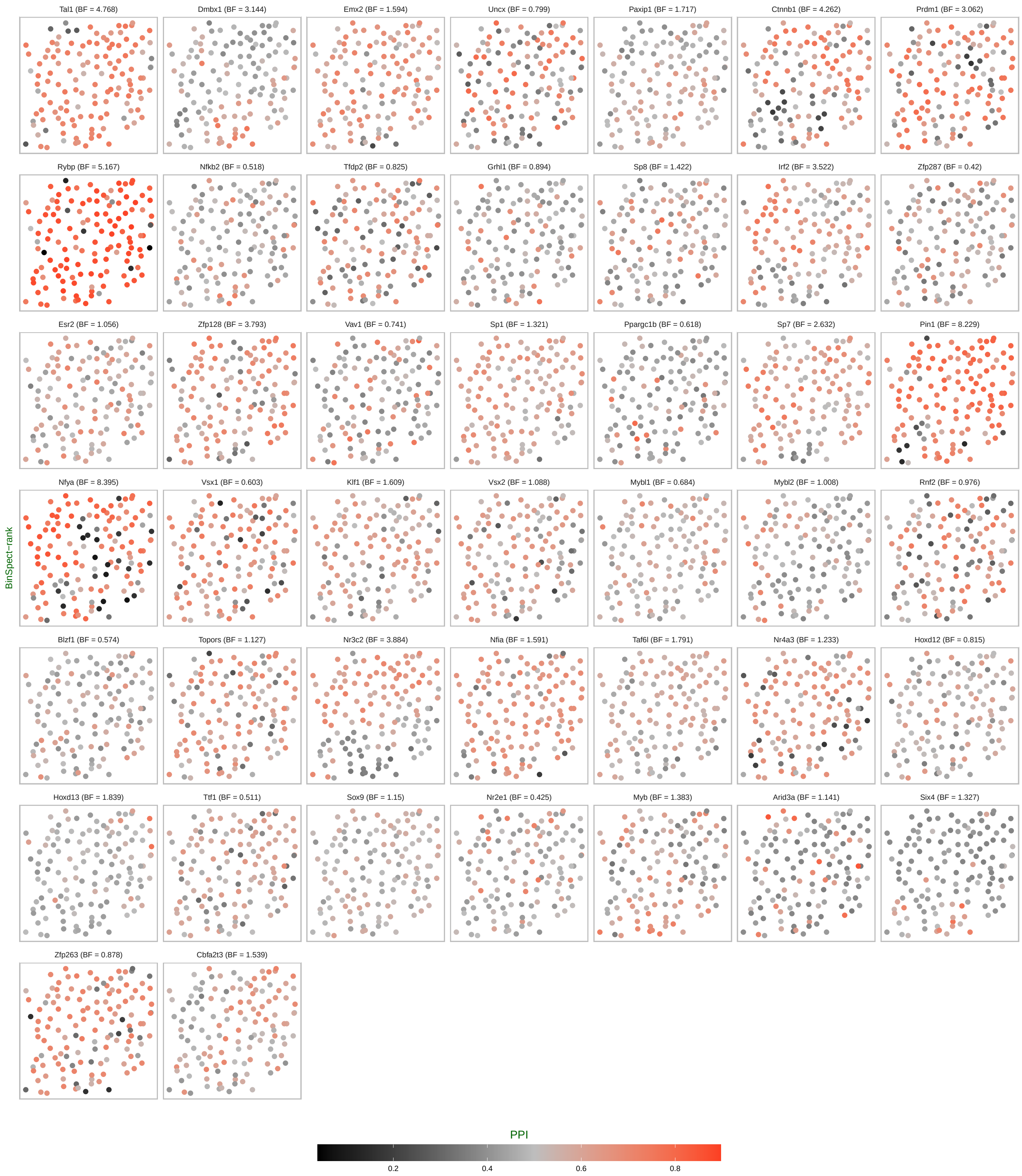

Figure S7: Mouse hippocampus seqFISH data: Spatial pattern of hidden gene expression indicator of SVGs detected by BinSpect-rank.

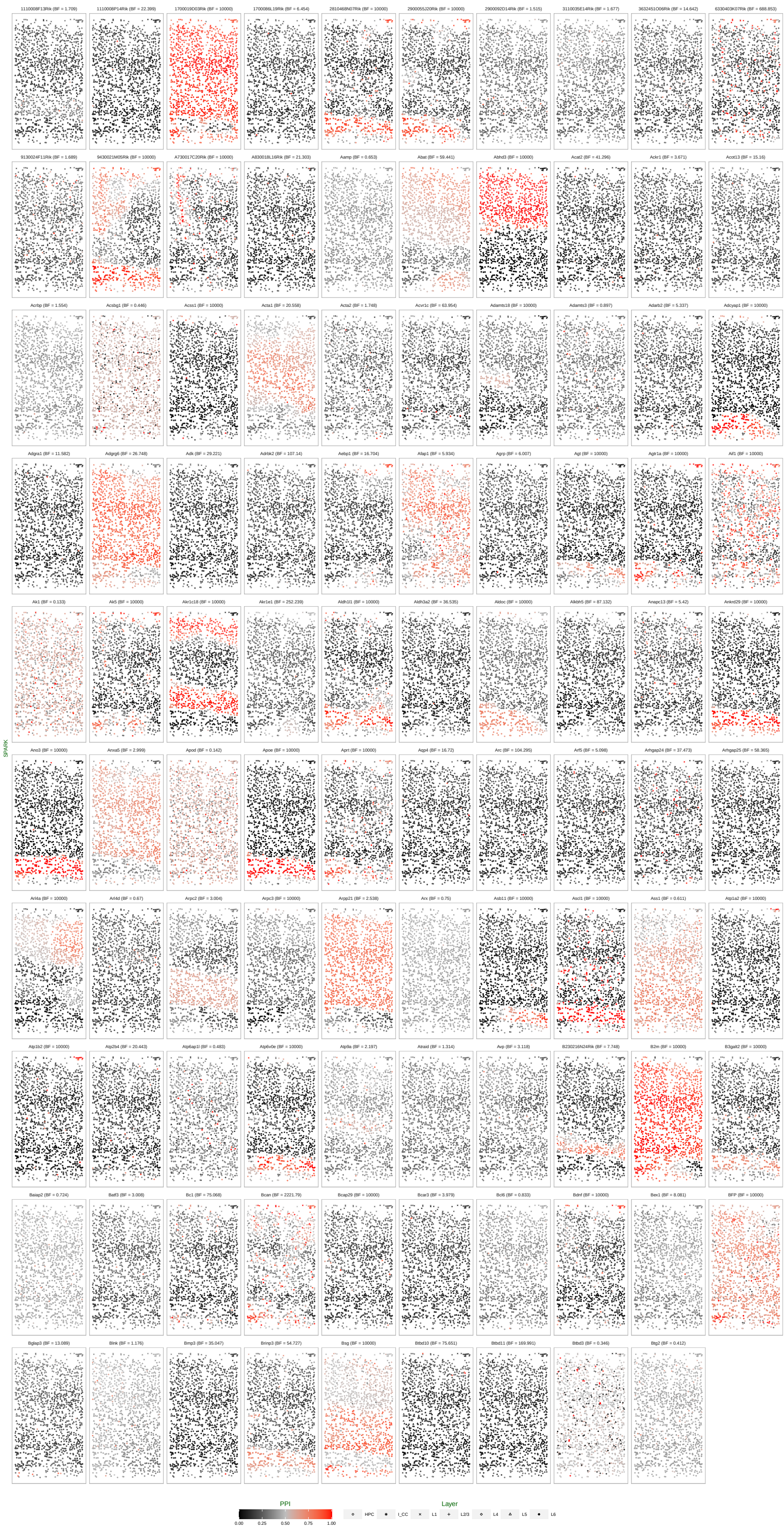

Figure S8: Mouse visual cortex STARmap data: Spatial pattern of hidden gene expression indicator of SVGs detected by SPARK.

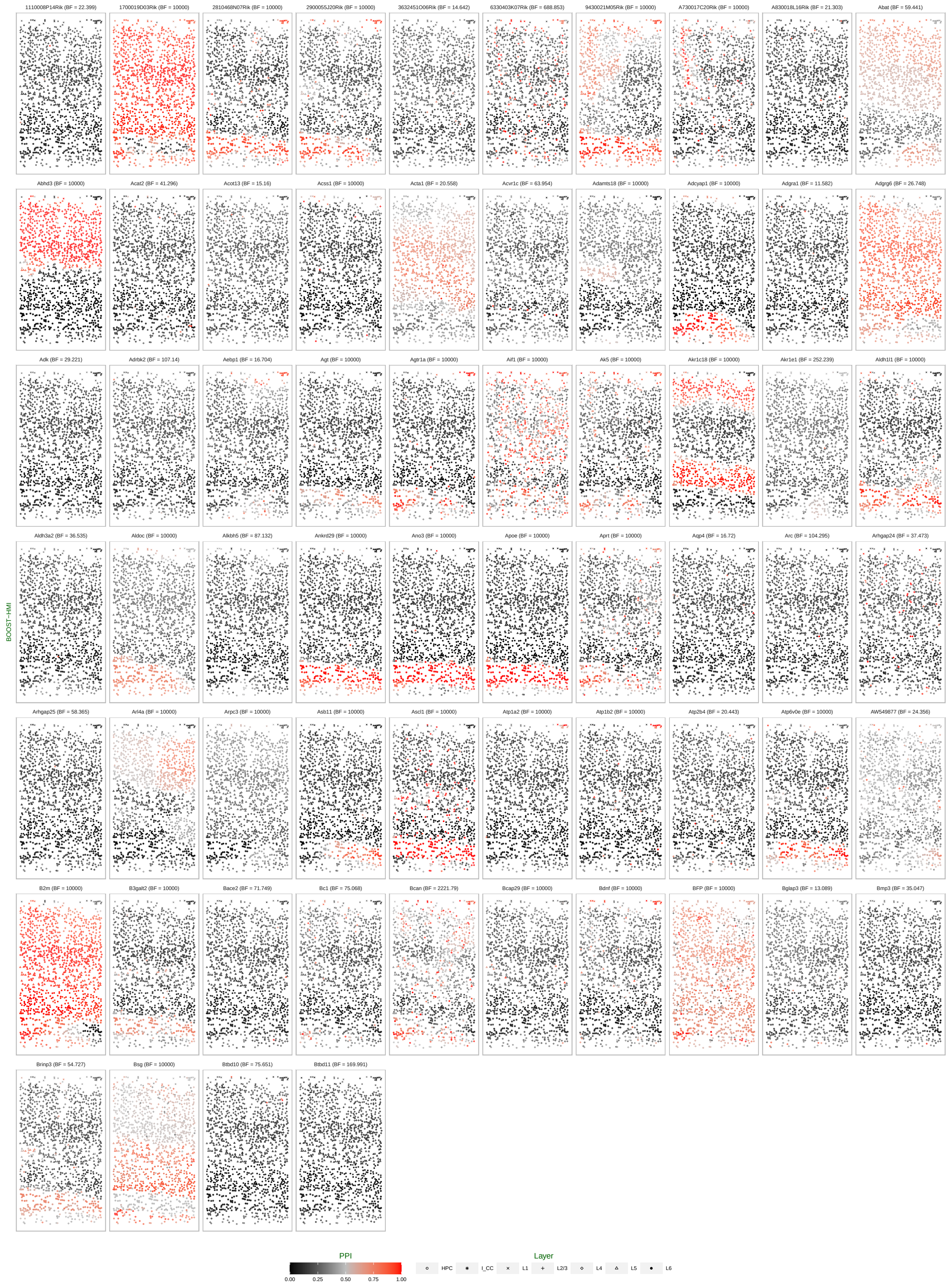

Figure S9: Mouse visual cortex STARmap data: Spatial pattern of hidden gene expression indicator of SVGs detected by BOOST-HMI.

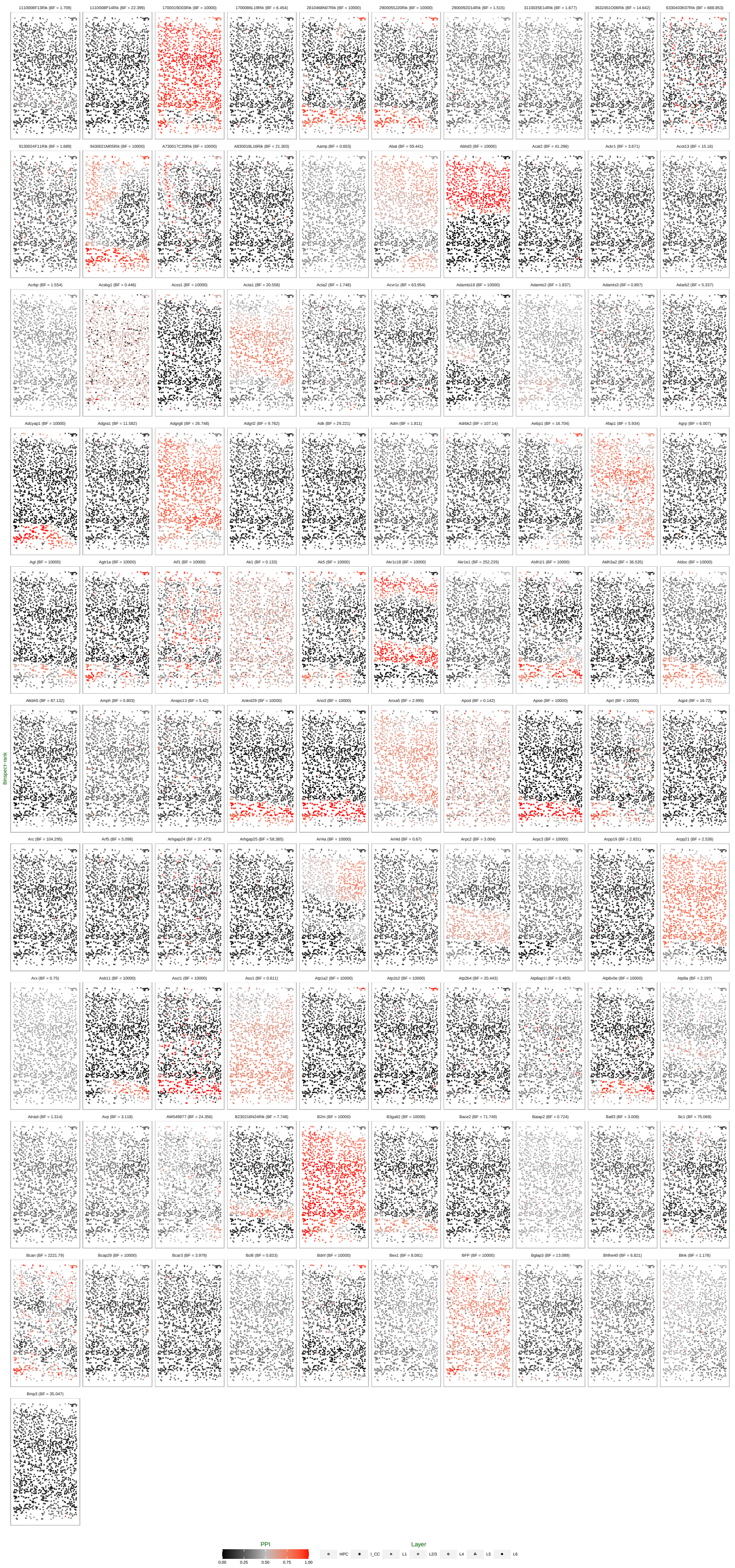

Figure S10: Mouse visual cortex STARmap data: Spatial pattern of hidden gene expression indicator of SVGs detected by BinSpect-rank.

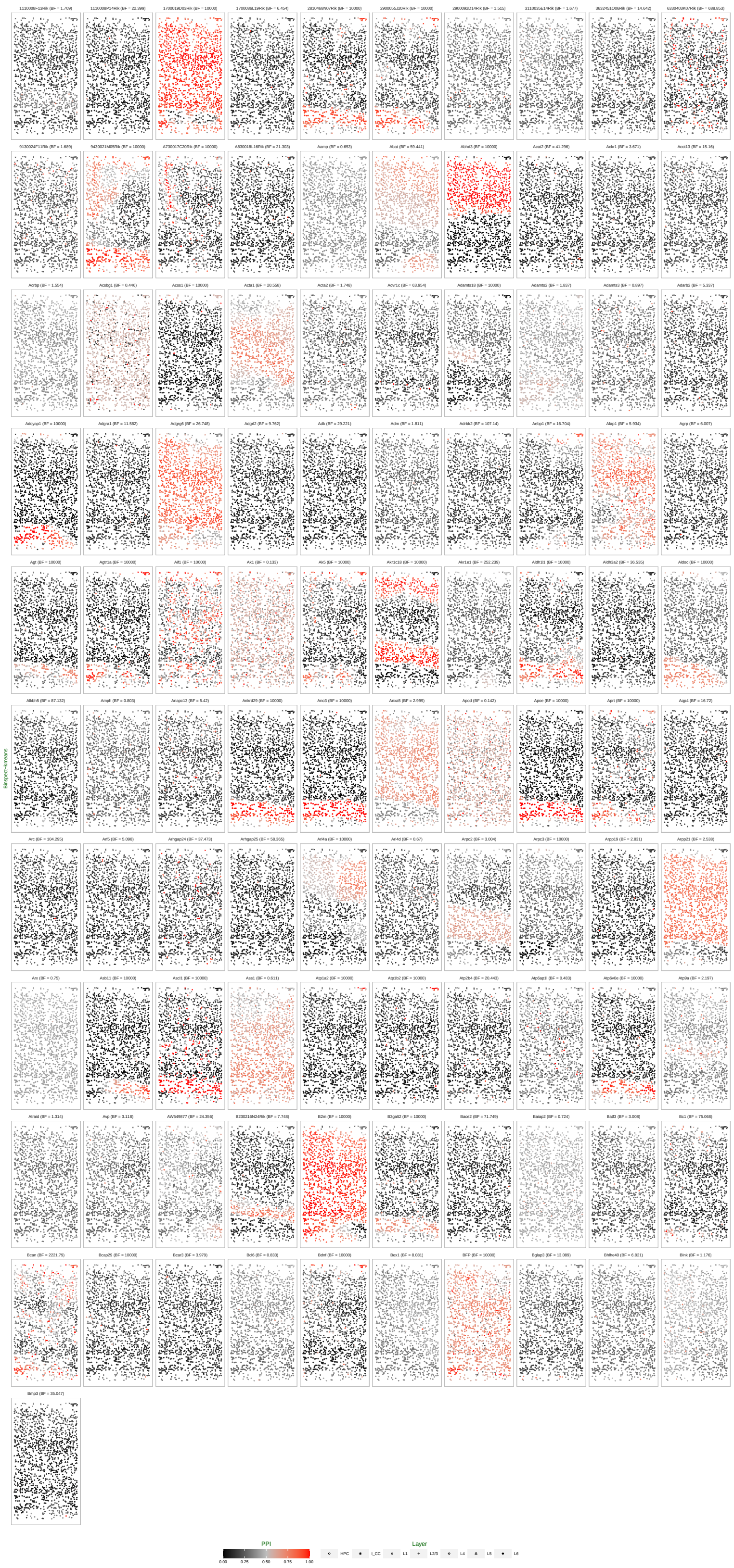

Figure S11: Mouse visual cortex STARmap data: Spatial pattern of hidden gene expression indicator of SVGs detected by BinSpect-kmeans.

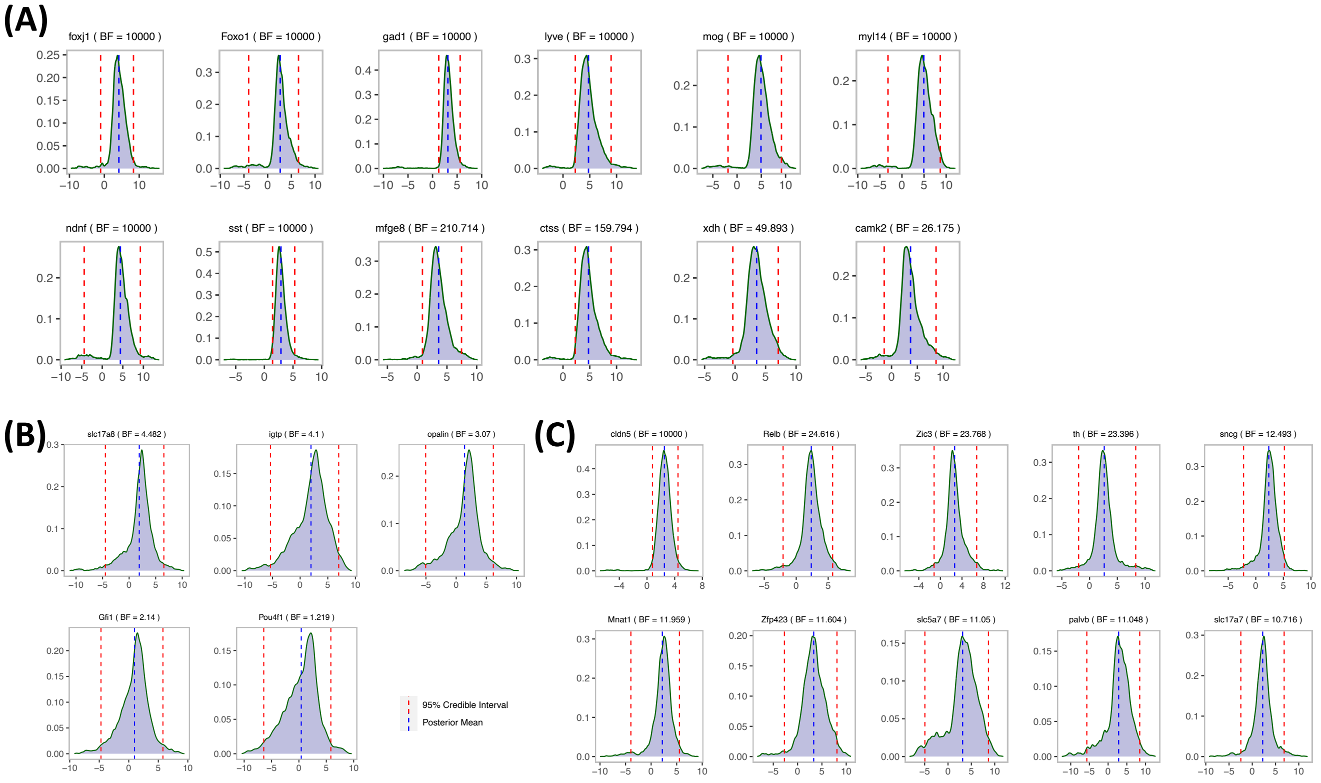

Figure S12: Mouse hippocampus seqFISH data: (A) Posterior distribution of $\boldsymbol{\theta}$ of the 12 SVGs detected by both SPARK and BOOST-HMI. (B) Posterior distribution of $\boldsymbol{\theta}$ of the five SVGs detected by SPARK only. (C) Posterior distribution of $\boldsymbol{\theta}$ of the ten SVGs detected by BOOST-HMI only. The three figures correspond to panels (A)(B)(C) in Figure 2.

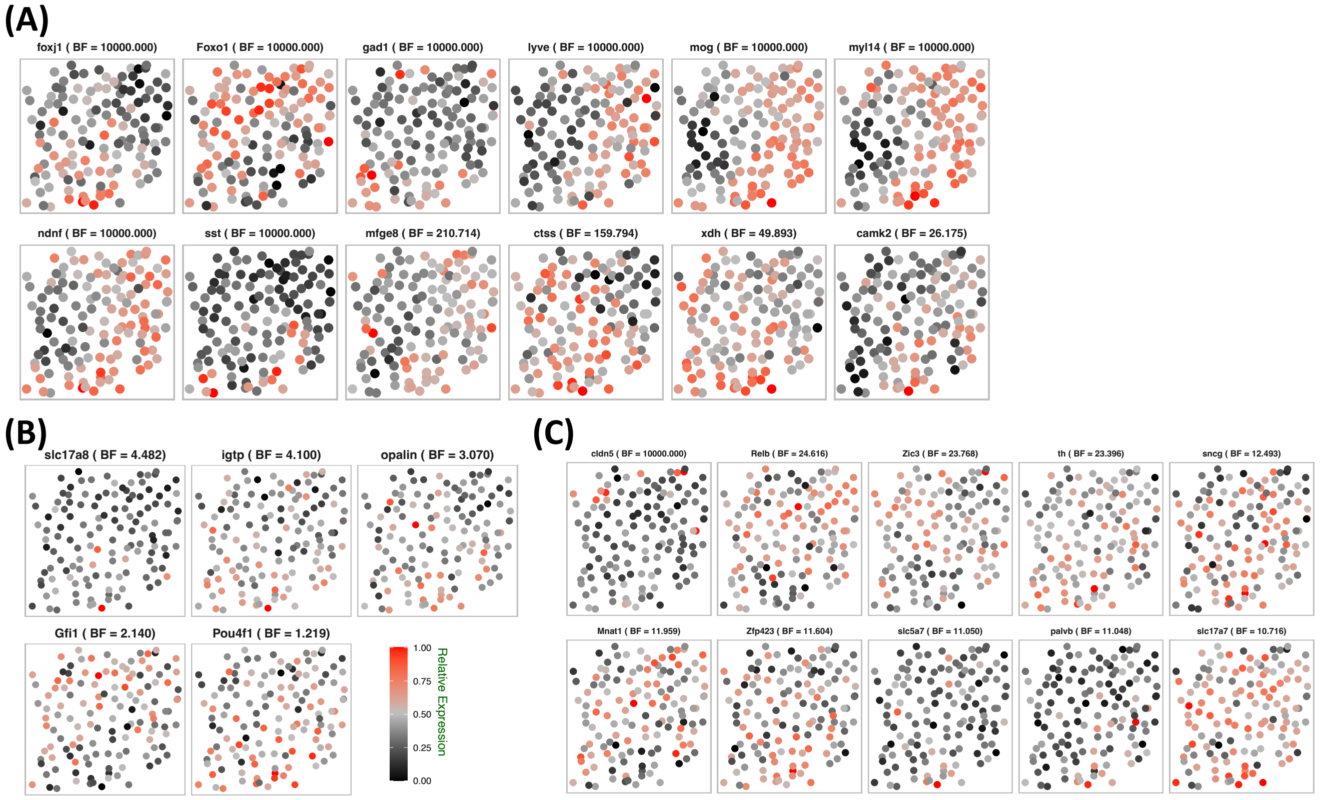

Figure S13: Mouse hippocampus seqFISH data: (A) Spatial distribution of relative gene expressions of the 12 SVGs detected by both SPARK and BOOST-HMI. (B) Spatial distribution of relative gene expressions of the five SVGs detected by SPARK only. (C) Spatial distribution of relative gene expressions of the ten SVGs detected by BOOST-HMI only. The three figures correspond to panels (A)(B)(C) in Figure 2.

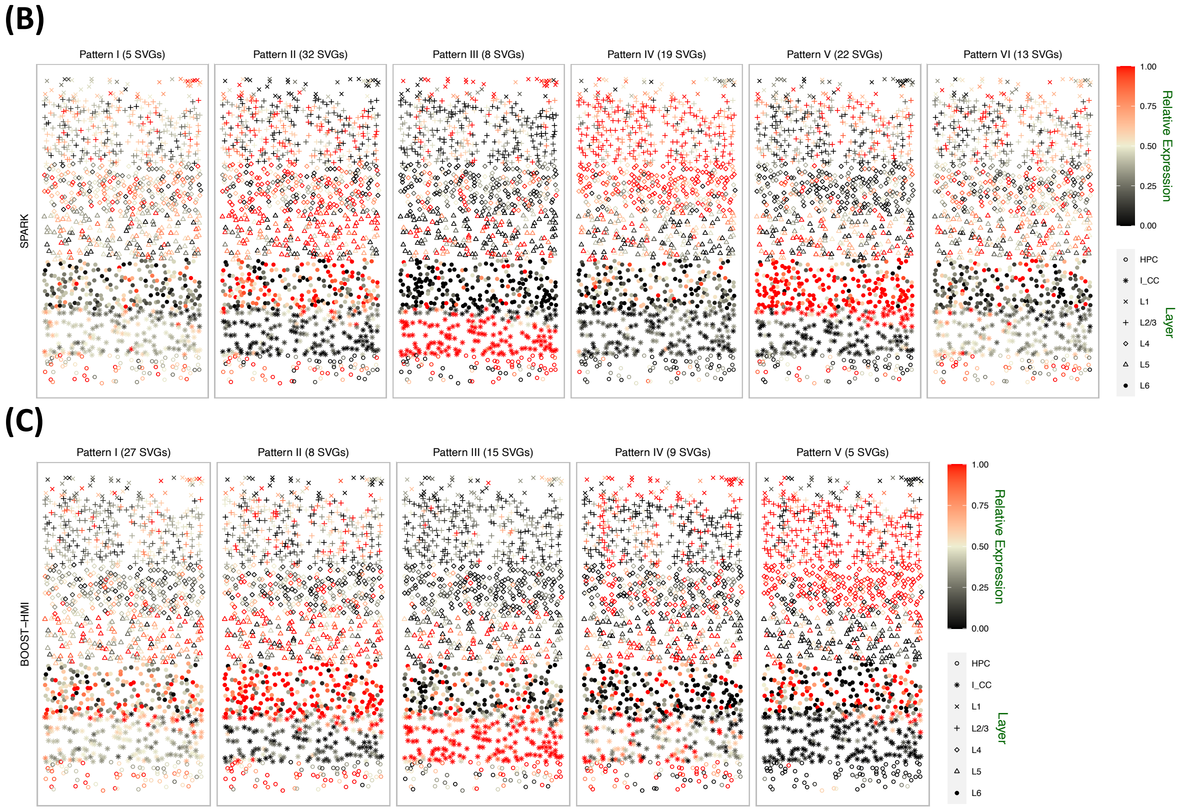

Figure S14: Mouse visual cortex STARmap data: (B) Spatial distribution of relative gene expression of the six SVG patterns detected by SPARK. (C) Spatial distribution of the relative gene expression of the five SVG patterns detected by BOOST-HMI. These two figures correspond to panels (B) and (C) in Figure 3.

Table S1. A hierarchical formulation of BOOST-HMI for identifying SV genes.

Hierarchical model:

$$y_{ij}|\xi_{ij},\eta_{ij},\mu_{0j},\mu_{1j},\phi_{0j},\phi_{1j} \sim \begin{cases} \text{NB}(y_{ij};\mu_{1j},\phi_{1j})^{\xi_{ij}}\text{NB}(y_{ij};\mu_{0j},\phi_{0j})^{1-\xi_{ij}} & \eta_{ij}=0 \\ I(y_{ij}=0) & \eta_{ij}=1 \end{cases}$$

Negative bionomial priors:

$$\mu_{0j},\mu_{1j} \sim \text{Ga}(a_\mu,b_\mu)$$

$$\phi_{0j},\phi_{1j} \sim \text{Ga}(a_\phi,b_\phi)$$

False zero indicator prior:

$$\eta_{ij}|\pi_i \sim \text{Bern}(\pi_i)$$

$$\pi_i \sim \text{Be}(a_\pi,b_\pi)$$

Bayesian Hidden mark interaction model:

$$V(\boldsymbol{\xi}_j|\omega_{0j},\omega_{1j},\theta_j) = \omega_{0j}\sum_i I(\xi_{ij}=0) + \omega_{1j}\sum_i I(\xi_{ij}=1) + \sum_{i\sim i'} I(d_{ii'}<c_d)\theta_j\exp(-\lambda_0 d_{ii'})I(\xi_{ij}\neq\xi_{i'j})$$

Energy function priors:

$$\omega_{0j} \sim \text{N}(\mu_\omega,\tau_\omega^2)$$

$$\theta_j \sim \text{N}(\mu_\theta,\tau_\theta^2)$$

Fixed hyperparameters:

$$a_\pi,b_\pi,a_\mu,b_\mu,a_\phi,b_\phi,\mu_\omega,\tau_\omega,\mu_\theta,\tau_\theta$$

Table S2. Computation time in seconds for analyzing the two real datasets. Computation were conducted using a MacBook M1 with 3.2GHZ 8-core processor and 8GB memory. For BOOST-HMI, the reported computation time represents the execution time per 1,000 MCMC iterations.

Data set	# of cells (n)	# of genes (p)	BOOST-HMI	SPARK	BinSpect-kmean	BinSpect-rank
Mouse hippocampus seqFISH data	131	249	51.5	32.8	8.2	5.4
Mouse visual cortex STARmap data	1523	107	11232.1	120.4	10.7	7.8

REFERENCES

Jiang, X., Xiao, G., and Li, Q. (2022). A Bayesian modified ising model for identifying spatially variable genes from spatial transcriptomics data. *Statistics in Medicine* 41, 4647–4665. doi:https://doi.org/10.1002/sim.9530
